# Hypothyroidism Side Effect in Patients Treated with Sunitinib or Sorafenib: Clinical and Structural Analyses

**DOI:** 10.1371/journal.pone.0147048

**Published:** 2016-01-19

**Authors:** Mao Shu, Xiaoli Zai, Beina Zhang, Rui Wang, Zhihua Lin

**Affiliations:** 1 School of Pharmacy and Bioengineering, Chongqing University of Technology, Chongqing 400054, China; 2 School of Chemistry and Chemical Engineering, Chongqing University, Chongqing 400044, China; University Campus Bio-Medico, ITALY

## Abstract

Tyrosine kinase inhibitors (TKIs) provide more effective targeted treatments for cancer, but are subject to a variety of adverse effects, such as hypothyroidism. TKI-induced hypothyroidism is a highly complicated issue, because of not only the unrealized toxicological mechanisms, but also different incidences of individual TKI drugs. While sunitinib is suspected for causing thyroid dysfunction more often than other TKIs, sorafenib is believed to be less risky. Here we integrated clinical data and *in silico* drug-protein interactions to examine the pharmacological distinction between sunitinib and sorafenib. Statistical analysis on the FDA Adverse Event Reporting System (FAERS) confirmed that sunitinib is more concurrent with hypothyroidism than sorafenib, which was observed in both female and male patients. Then, we used docking method and identified 3 proteins specifically binding to sunitinib but not sorafenib, i.e., retinoid X receptor alpha, retinoic acid receptors beta and gamma. As potential off-targets of sunitinib, these proteins are well known to assemble with thyroid hormone receptors, which can explain the profound impact of sunitinib on thyroid function. Taken together, we established a strategy of integrated analysis on clinical records and drug off-targets, which can be applied to explore the molecular basis of various adverse drug reactions.

## Introduction

Conventional chemotherapies usually do not discriminate tumor cells and other rapidly dividing normal cells, thus leading to unpredictable cytotoxic side effects. In contrast, targeted therapies specifically interfere with molecular targets located in tumor cells or involved in tumor growth. Therefore, targeted therapies may be particularly effective to those patients not reactive to cytotoxic agents. Also, targeted therapies may reduce certain toxicity related to cytotoxic agents (although causing other adverse effects as introduced below) [1]. Tyrosine-kinase inhibitors (TKIs) are a class of drugs mainly used in cancer treatment as targeted therapy [2]. Being analogues of ATP, TKIs typically compete with the ATP for binding site of particular oncogenic tyrosine kinases. By blocking the signaling pathways involved in the phosphorylation of many key proteins in signal transduction cascades, TKIs can depress tumor cell survival and proliferation. Imatinib is the first TKI to be introduced into clinical oncology, which is followed by novel ones, including sorafenib [3] and sunitinib [4]. Sorafenib is an orally active multi-kinase inhibitor that is approved for the treatment of renal cell carcinoma, hepatocellular carcinoma and some other types of cancer. It inhibits several tyrosine protein kinases (e.g., VEGFR and PDGFR) and Raf kinases [5]. Sunitinib is another oral and multi-targeted TKI. It is approved by the FDA for the treatment of renal cell carcinoma, also as a second-line therapy for patients whose gastrointestinal stromal tumor is resistant to imatinib. Tyrosine kinases inhibited by sunitinib include VEGFR, PDGFR, KIT, FLT3, etc [6].

With the increased use of TKIs for cancer treatment, a variety of unusual adverse effects begin to emerge [7]. It is a puzzling phenomenon that while the TKIs achieve therapeutic effects through similar mechanisms of action, on the other hand, they differ dramatically in certain adverse effects. A typical example is TKI-induced hypothyroidism (i.e., deficiency of thyroid hormone), which is characterized by a number of symptoms, such as tiredness, poor ability to tolerate cold, and weight gain. Sunitinib, for instance, is suspected for causing thyroid dysfunction more often than other TKIs [8]. In contrast, the incidence of hypothyroidism in sorafenib user is reported to be much lower [9–11]. The possible toxicological difference between sunitinib and sorafenib raises our attention, because they may serve as a precious pair of case and control drugs. Since these two drugs are broadly similar in their actions, except for hypothyroidism side effect, if some minor differences are found, they are highly likely to be associated with the pathogenesis of the drug-induced hypothyroidism.

In the present study, we primarily retrieved a large number of clinical reports of patients using sunitinib or sorafenib (Table 1) from FDA Adverse Event Reporting System (FAERS), in order to calculate the frequency of hypothyroidism adverse events. In the United States, the FDA is the major agency that responsibie for monitoring the safety of marketed drug products. For this purpose, FDA has established FAERS to collect and restore reports of adverse events, which are mostly sent by healthcare professionals and patients directly to the FDA on a voluntary basis. Those reports suggesting significant signals of certain adverse events can raise special attention of not only FDA safety evaluators, but also researchers worldwide [12–14]. FDA used to release the data to the public quarterly, but an analysis requires researchers to manually download and preprocess the data for every quarter starting from 2004. In an effort to provide easier access to FAERS, the openFDA initiative (https://open.fda.gov/) is launched in June 2014, which gives the official API access to the raw data of adverse events reports in a structured and computer-readable format. In the present study, therefore, we were enabled to efficiently identify the significant difference between sunitinib and sorafenib.

**Table 1:** Combined summary of sunitinib- and sorafenib-associated reports of adverse events.

		Sunitinib	Sorafenib
	Total	17242	9634
Sex	Female	5351 (31.03%)	2488 (25.83%)
	Male	10153 (58.89%)	6411 (66.55%)
	N/A	1738 (10.08%)	735 (7.63%)
Reproting Source	Health Professionals	11692 (67.81%)	7009 (72.75%)
	Consumers	4597 (26.66%)	1116 (11.58%)
	Lawyers	3 (0.02%)	1 (0.01%)
	N/A	950 (5.51%)	1508 (15.65%)

In addition, the toxicological mechanisms of TKI-induced hypothyroidism remain largely unknown. Since sunitinib and sorafenib are proved to differ in the risk of causing hypothyroidism, we hypothesized that if a certain protein tends to be unexpectedly targeted by sunitinib but not sorafenib, that protein may serve as a sunitinib-specific off-target and a candidate mediator of sunitinib-induced hypothyroidism. Even it is unaffordable to experimentally search for such kind of off-targets among a great number proteins, we alternatively used the DRAR-CPI online server [15], which provided a free but highly efficient platform for *in silico* simulation of molecular structures and prediction of preferred orientation of drug-protein bindings. By examining the differences between sunitinib and sorafenib in their interaction profile towards a series of human proteins, several candidates were successfully highlighted, which may be associated with the underlying mechanisms of hypothyroidism side effect.

## Results

### Sunitinib co-occured with hypothyroidism events more frequently than sorafenib

Although sunitinib, as compared with sorafenib, has been suspected for higher risk of inducing hypothyroidism, the current clinical evidences were only based on relatively small samples with limited persuasiveness [9–11]. On the other hand, FAERS provided us with a unique opportunity to validate previous observations with a large sample of patients. We retrieved all the adverse events related to sunitinib and sorafenib, respectively (see Methods). To avoid the complication of drug-drug interaction, the patients using sunitinib and sorafenib simultaneously were excluded, thus leaving 17242 cases of sunitinib and 9634 of sorafenib. Hypothyroidism adverse reaction was reported in 1.66% of the sunitinib-related cases, while the same problem was found in only 0.57% of the sorafenib-related cases (Table 2). The one-tailed Fisher’s exact test was performed to compare these two drugs, and the result suggested that the hypothyroidism reporting rate was significantly higher for sunitinib than sorafenib (P = 2.86E-16). Hence, FAERS data corroborated the specific association between hypothyroidism and sunitinib.

**Table 2:** Test for the difference of the hypothyroidism reporting rate between sunitinib and sorafenib in the FDA adverse event reporting system (FAERS).

	Sunitinib	Sorafenib
Hypothyroidism Reported	287	55
Hypothyroidism Not Reported	16955	9579
Ratio of Hypothyroidism Report (%)	1.66	0.57
Odds Ratio (95% CI)	2.95 (2.21 − 3.94)	
P-value*	2.86E-16	

* One-tailed Fisher’s exact test was performed for the higher rate of hypothyroidism for sunitinib compared to sorafenib.

### Higher risk of sunitinib than sorafenib regardless of sex factor

Gender difference in thyroid system function is a common medical knowledge [16]. Because of that, the etiology and prevalence of hypothyroidism naturally differ between female and male population, i.e., women are observed to be more affected by hypothyroidism than men [17–20]. Although drug-induced hypothyroidism is a type of side effect rather than a primary disease, in the context of this study, we are still cautious about the possible confounding effect of sex factor on the FAERS statistics. Therefore, we further compared the sunitinib and sorafenib for the reporting rate of hypothyroidism, with the female and male patients separated. As suggested by the results (Table 3), sunitinib co-occurred with hypothyroidism events more frequently in both female and male patients. Even though the reporting odds ratio between sunitinib and sorafenib was higher in women than men, it did not suggest any significant sex difference (P > 0.1 for multivariate logistic regression, data not shown). Thus, we confirmed that the difference in hypothyroidism incidence between sunitinib and sorafenib users should be independent of sex factor and be mainly attributed to the unique toxicity of sunitinib.

**Table 3:** The difference of the hypothyroidism reporting rate between sunitinib and sorafenib regarding female and male populations.

	Female		Male	
	Sunitinib	Sorafenib	Sunitinib	Sorafenib
Hypothyroidism Reported	119	18	138	35
Hypothyroidism Not Reported	5232	2470	10015	6376
Ratio of Hypothyroidism Report (%)	2.22	0.72	1.36	0.55
Odds Ratio (95% CI)	3.12 (1.90 − 5.14)		2.51 (1.73 − 3.64)	
P-value*	3.32E-07		1.28E-07	

* One-tailed Fisher’s exact test was performed for the higher rate of hypothyroidism for sunitinib compared to sorafenib.

### Differences in target binding profile between sunitinib and sorafenib

**Fig 1:**
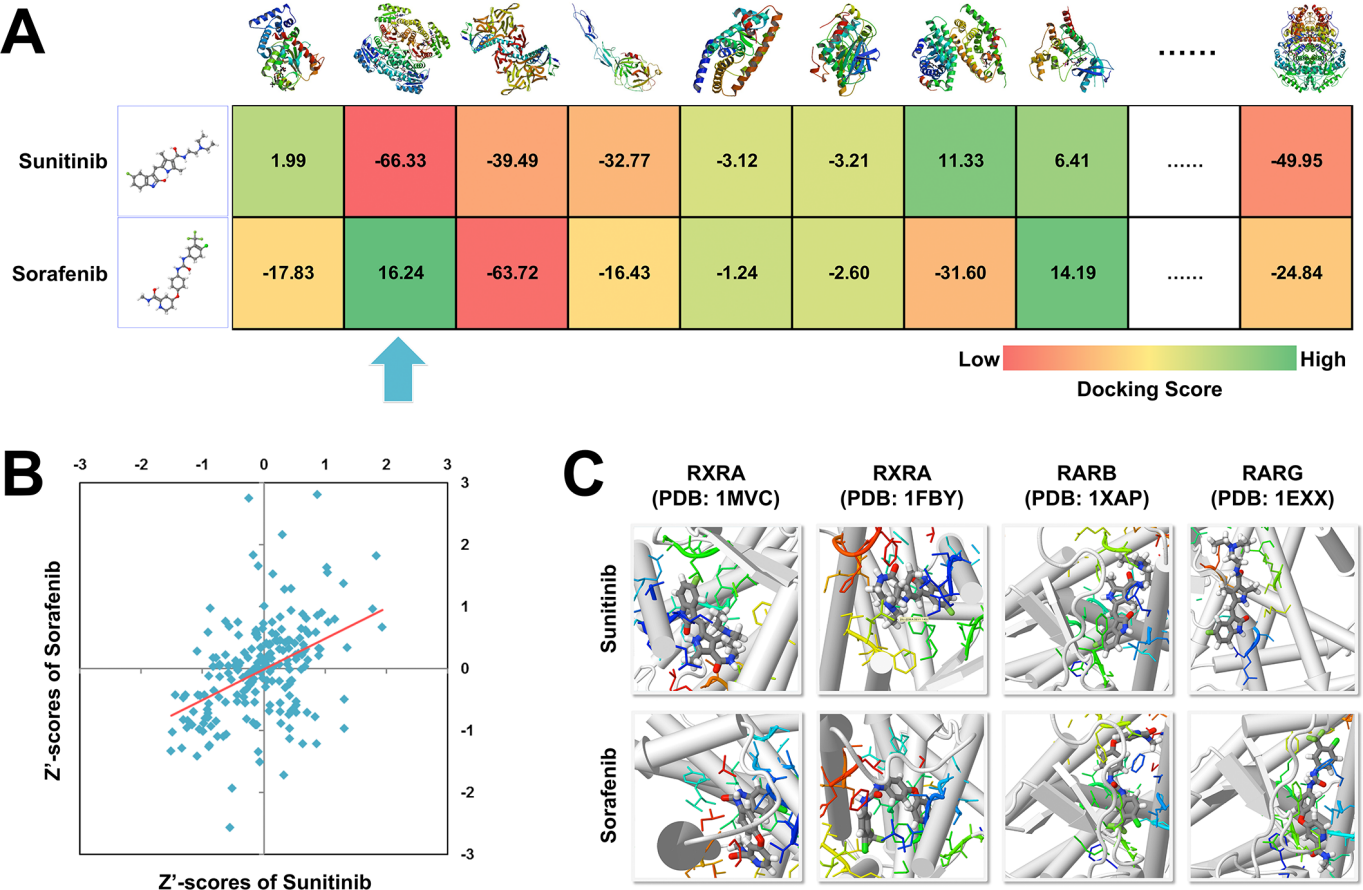
Structural comparison of sunitinib and sorafenib towards potential off-targets. (A) The rationale of the comparison between sunitinib and sorafenib. Simulated binding affinity is measured by docking scores. We pay particular attention to those targets (as indicated by the arrow) showing much higher affinity with sunitinib than with sorafenib. (B) The comparability between sunitinib and sorafenib is verified by their positively correlated Z’-score vectors. (C) The whole molecule of sunitinib binds deep into the pockets of retinoic acid receptors (RXRA has two structure models in Protein Data Bank for docking, while RARB and RARG have one). In contrast, the sorafenib molecule is less well accommodated in the pockets due to steric hindrance in different directions. As suggested by docking scores, the binding strengths of sunitinib towards retinoic acid receptors tend to be higher than those of sorafenib. The figures are produced by DRAR-CPI server.

The purpose of the above pharmacovigilance data analysis was not only to corroborate the relatively higher hypothyroidism risk of sunitinib, but more importantly to understand underlying toxicological mechanisms. Due to the complex nature of drug toxicity and limited knowledge about drug-induced hypothyroidism, it has always been extremely difficult to perform hypothesis-driven study on the detailed toxicological mechanisms. Because of that, we alternatively adopted a data-driven strategy and explored the potential off-targets specifically interacting with sunitinib but not sorafenib (Fig 1A), by using chemical-protein docking method [21,22] (see Methods). For this purpose, we uploaded the molecular structure information [23] of sunitinib and sorafenib into the DRAR-CPI server. The interaction strength of a drug molecule to a protein was primarily measured by docking score (i.e., the lower docking score, the stronger strength of binding). In addition, considering the relative strength based on the interaction matrix of a collection of drugs and proteins restored in DRAR-CPI, the raw docking score was further normalized into Z'-score, so as to address the endogenous variance among proteins or chemicals and increase data accuracy [24].

A total of 208 protein pockets were suitable for docking with both sunitinib and sorafenib molecues (S1 Dataset). We primarily calculated the Pearson’s correlation coefficients (PCC) between Z’-score vectors of sunitinib and sorafenib across these 208 targets (Fig 1B). As two highly comparable drugs of the TKI class, sunitinib and sorafenib exhibited positively correlated protein binding profiles (PCC = 0.43, p = 1.30E-10), which underlined their overall similarity in target spectrum and pharmacology. We therefore hypothesized that the minor distinctions in protein binding affinity between them might account for the sunitinib-biased risk of hypothyroidism. Following this hypothesis, we further calculated the Z’-score difference between sunitinib and sorafenib towards all the proteins, in order to search for the targets with higher affinity (i.e., lower Z’-score) to sunitinib. Then, we performed permutations for each target by randomly selecting two Z’-scores of sunitinib and sorafenib and measuring their difference 10,000 times. Finally, a p-value was obtained as the one-tailed probability when the random values were lower than the difference between sunitinib and sorafenib (see Methods).

Targets with permutation p-value less than the 0.05 cutoff are shown in Table 4, among which the retinoic acid receptors, including retinoic acid receptor RXR-alpha (RXRA), retinoic acid receptor beta (RARB) and retinoic acid receptor gamma (RARG), raise our particular attention. Thyroid hormone activates thyroid hormone receptors, a type of nuclear receptor, which further regulate gene expression by binding to hormone response elements (HREs) in DNA either as monomers, homodimers or heterodimer with other nuclear proteins [25]. It has been repeatedly reported that retinoic acid receptors, in particular, serve as actively heterodimer partners for thyroid hormone receptors [26–28]. Showing significantly lower docking Z’-scores towards sunitinib than sorafenib, the retinoic acid receptors specifically provide sunitinib with pockets to deeply fit in (Fig 1C). Given such differences, we hypothesize that sunitinib may profoundly interfere in the function of retinoic acid receptors and disturb thyroid hormone signals (as discussed bellowed).

**Table 4:** Targets with significantly lower docking Z’-scores towards sunitinib than sorafenib.

PDB ID	Putative Target	Sunitinib Z'-Score	Sorafenib Z'-Score	Difference	P-value
1XAP	Retinoic acid receptor beta	-0.237588	2.74816	-2.985748	0.0041
1MVC	Retinoic acid receptor RXR-alpha	-0.171023	1.83455	-2.005573	0.0293
1EXX	Retinoic acid receptor gamma	0.883029	2.81209	-1.929061	0.0324
1RFN	Coagulation factor IX	0.309693	2.16421	-1.854517	0.0383
1Z6J	Coagulation factor VII	-0.867828	0.979206	-1.847034	0.0388
1FBY	Retinoic acid receptor RXR-alpha	-0.0783559	1.6608	-1.7391559	0.0478

## Discussion

Advances in our understanding of cancer biology lead to the development and approval of various TKI anticancer drugs, such as sunitinib than sorafenib. In the meantime, large-scale data regarding potential safety liability remain rare, but urgently needed to offer alternative treatments to specific patient groups when adverse effect seems unfavorable and intolerable. In particular cases, hypothyroidism refers to a common disease of the endocrine system, which may be caused by low iodine diet [29], autoimmune effects [30] and other factors. Hypothyroidism, as a drug side effect, is uncommonly complicated, because drugs may interfere at different steps in thyroid hormone homeostasis [31,32], including synthesis and secretion from the thyroid gland, thyroid hormone metabolism, absorption of thyroid hormone (especially in patients subject to pre-existed hypothyroidism), etc. While drug induced hypothyroidism is not observed in clinical trials very often, in several independent studies with relatively small samples, unexpected high frequencies of hypothyroidism are found among patients accepting sunitinib treatment [33–36]. This observation gives us strong incentive to confirm the thyroid effect of sunitinib with clinical data in larger scale. More importantly, from the perspective of drug off-targets, we intend to provide an innovative solution to addressing the complexity of thyroid dysfunction and understanding the toxicological mechanisms of sunitinib.

Currently, clinical trials through phase I to III remain the major checkpoints for detecting and preventing potential adverse drug reactions. However, because of the limited number of patients involved and the short duration of the study, some drugs with certain safety liabilities may still be overlooked and approved for marketing [37,38]. Thus, post-marketing pharmacovigilance that begins when the drug enters the general market can play a significant role in early identification and retrospective investigation of overlooked adverse drug events. In the present study, we realized that the large-scale clinical reports documented in FAERS could provide a unique opportunity to consolidate the suspected difference in thyroid toxicity between sunitinib and sorafenib. In addition, despite of the potential complications of sex factor, the higher reporting frequency of hypothyroidism among patients using sunitinib than those using sorafenib proved to be sex-independent. Despite the great value of FAERS data, there are some limitations inherent to its spontaneous nature. For example, all reports in FAERS are related to drug users affected by certain side effects, without healthy or unaffected people as control subjects. Thus, rather than using control subjects, we alternative defined sorafenib as a ‘reference drug’, which was directly compared with sunitinib (as the ‘case drug’). In this way, the problem caused by the lack of control was at least partly avoided. Also, it is important to better understand the safety risks of the combination thyroid cancer therapy. Unfortunately, the number of adverse event reports involving multiple drugs in FAERS data was too low to achieve an acceptable statistical power. Therefore, we suggest that other clinical data regarding the combined treatment should be scrutinized to address this issue. But it should be noted that, although the FDA’s adverse events data were used for this study, the results and conclusions of this article were solely those of the authors and should not represent the opinions of FDA.

By now, several hypotheses have been proposed to explain TKI-induced hypothyroidism, including reduced synthesis of thyroid hormones [33], inhibition of thyroid uptake of iodine [39], atrophy of the thyroid owing to drug toxicity [36], preceding thyroiditis with associated transient thyrotoxicosis [40], and immune dysregulation [41]. However, these theories have yet to be confirmed. More importantly, the mechanism responsible for the development of hypothyroidism in patients treated with sunitinib is still not clearly explained [42,43], suggesting that there can be some previously unknown mechanism of thyroid toxicity. We therefore use DRAR-CPI to explore the potential off-targets for sunitinib. A unique opportunity to addressing this issue is the pharmacological reference provided by sorafenib. According to the guilt-by-association principle, since sunitinib and sorafenib are largely similar in therapeutic effects, their difference in off-target binding may be directly correlated to the hypothyroidism side effect specific to sunitinib. Interestingly, retinoic acid receptors seem to be more probably to interact with sunitinib than with sorafenib. Based on this observation, we propose the following hypothesis. It is well known that thyroid hormone action is mediated by the heterodimers of nuclear thyroid hormone receptors and retinoid acid receptors [27]. Sunitinib may compete with thyroid hormone receptors for the opportunity of binding with retinoic acid receptors, thus deteriorating the dysregulation of thyroid hormone due to either carcinogenesis [44] or radiation therapy [45]. The off-target hypothesis generated in this study can represent candidates for wet-lab toxicological research and biomarker development at genotype and gene expression level in TIK therapy. However, it must be kept in mind that the 208 drug targets covered by DRAR-CPI are only a limited representation of the entire human proteome, leaving numerous targets currently unexamined. Because of that, we expect the CPI servers and other docking tools to evolve and support systematical study on more off-targets in the future.

Despite the progress shown in this study, we believe some improvements and extensions will be necessary and important to better understanding TKI-induced thyroid disorders. First, after binding with protein targets, drug molecules must disturb the expression of certain genes to produce either desirable or unwanted responses. Here we have explored the information about target binding and clinical responses, but in subsequent studies, drug-related genomic expression data at mRNA [46], microRNA [47], metabolic [48] or network [49,50] level should also be examined, so as to further identify the signal pathways involved in hypothyroidism side effect. Second, to further understand drug toxicity at the personalized level, we would explore how the mutations of relevant genes may affect the drug-target interaction [51]. Third, it has been reported by FDA that a higher dose should be a risk factor for drug-induced adverse reactions [52]. However, dosage information was missing in many FAERS reports, which prevented us to examine this aspect in the present study. So we suggest that the dosage factor should be taken into consideration in the subsequent clinical studies on TKIs, so as to further optimize the anti-cancer regimens. Fourth, impact of hypothyroidism (in terms of incidence and grade) may vary between different types of cancer, but this issue was not addressed in the presents study. The current results can warrant subsequent research on the confounding effect of different cancer pathologies. Fifth, besides sunitinib and sorafenib, various TKI drugs are currently used to treat cancer and may cause thyroid reactions. For instance, it has been reported that axitinib may induce thyroid dysfunction in clinical trials [53,54]. Therefore, we expect to apply our method to the adverse effects of axitinib and other TKIs in the future.

In summary, this study presented a practical and efficient strategy of investigating the underlying mechanisms of adverse drug reactions. Due to the complicated nature of *in vivo* drug action, there is usually no initial clue about the reasons of drug toxicity. Fortunately, there are many classes of drugs developed based on the same theory and showing highly similar pharmacological properties. If empirical observations suggest potential differences in adverse reactions between a test drug of interest and an analogous drug, the procedures demonstrated in this study will illuminate the toxicological mechanisms by, 1) helping confirm the difference in clinical toxicology between the test drug and the analogous drug; 2) predicting the off-target interaction specific to the test drugs. We believe this data-driven strategy would largely enhance the current experiment-based framework of toxicological research.

## Materials and Methods

### Statistical analysis of drug adverse events

The original reports were restored in the FDA Adverse Event Reporting System (FAERS) and queried through OpenFDA platform following the official tutorial (https://open.fda.gov/api/reference/). The events bearing either sunitinib or sorafenib and submitted from January 2004 to June 2014 were retrieved with the drug generic name “SUNITINIB MALATE” or “SORAFENIB”. However, the cases that related with both sunitinib and sorafenib were excluded. And for sex-specific analysis, those reports without patient sex information were not counted. With regard to the numbers of hypothyroidism events co-occurred with sunitinib (N_11_), non-hypothyroidism events co-occurred with sunitinib (N_10_), hypothyroidism events co-occurred with sorafenib (N_01_), non-hypothyroidism events co-occurred with sorafenib (N_00_), we calculated the odds ratio as (N_11_× N_00_)/ (N_10_× N_01_), in order to examine the difference in the frequency of hypothyroidism reporting. And one-tailed Fisher's exact test was performed to measure the significance level.

### Drug-protein docking

The chemical structures of sunitinib and sorafenib, as described by simplified molecular-input line-entry system (SMILES), were queried from the DrugBank database (http://www.drugbank.ca/) and input into the DRAR-CPI online server (http://cpi.bio-x.cn/drar/). Then DRAR-CPI constructed the *in silico* interaction between sunitinib or sorafenib and 208 protein pockets using the Docking method. DRAR-CPI automatically normalized the output Docking scores into Z’-scores, which were exported for subsequent analysis. We calculated the Z’-score difference between sunitinib and sorafenib towards protein i,

$$D_{i}^{Sun-Sor}=Z_{i,Sun}^{\prime }-Z_{i,Sor}^{\prime }$$ (1)

We also calculated the probability of the Z’-score difference lower than $D_{i}^{Sun-Sor}$ regarding two randomly selected proteins among all the 208 targets, which could be expressed as,

$$p_{i}=P\left(Z_{j,Sun}^{\prime }-Z_{k,Sor}^{\prime }<D_{i}^{Sun-Sor}\right)\;\left(j,k\in \left[1,208\right]\right)$$ (2)

The targets with *p*_*i*_ < 0.05 were highlighted.

## Supporting Information

S1 Dataset Docking scores of sunitinib and sorafenib towards 208 protein pockets. (XLSX)
